# Biomarkers for the Rich and Dangerous: Why We Ought to Extend Bioprediction and Bioprevention to White-Collar Crime

**DOI:** 10.1007/s11572-018-9477-6

**Published:** 2018-08-10

**Authors:** Hazem Zohny, Thomas Douglas, Julian Savulescu

**Affiliations:** Oxford Uehiro Centre for Practical Ethics, Faculty of Philosophy, University of Oxford, Suite 8, Littlegate House, 16–17 St Ebbes St., Oxford, OX11PT UK

**Keywords:** Biomarkers, Bioprediction, Bioprevention, Corporate psychopathy

## Abstract

There is a burgeoning scientific and ethical literature on the use of biomarkers—such as genes or brain scan results—and biological interventions to predict and prevent crime. This literature on biopredicting and biopreventing crime focuses almost exclusively on crimes that are physical, violent, and/or sexual in nature—often called blue-collar crimes—while giving little attention to less conventional crimes such as economic and environmental offences, also known as white-collar crimes. We argue here that this skewed focus is unjustified: white-collar crime is likely far costlier than blue-collar crime in money, health, and lives lost. Moreover, attempts to biopredict and bioprevent blue-collar crime may entail adopting potentially unfair measures that target individuals who are already socio-economically disadvantaged, thus compounding pre-existing unfairness. We argue, therefore, that we ought to extend the study of bioprediction and bioprevention to white-collar crime as a means of more efficiently and fairly responding to crime. We suggest that identifying biomarkers for certain psychopathic traits, which appear to be over-represented among senior positions in corporate and perhaps political organisations, is one avenue through which this research can be broadened to include white-collar crime.

## Introduction

There is a burgeoning scientific and ethical literature on biologically predicting and preventing crime. This literature typically investigates the use of biomarkers—such as genes or brain scan results—to predict the likelihood that an individual will engage or re-engage in criminal activities (e.g., Singh et al. 2013), and the use of biological interventions—such as brain-active drugs—to prevent an individual from offending or reoffending (e.g., Chew et al. 2018). These are important and controversial issues on which we believe progress is being made. However, in this article we wish to advance a criticism of the existing scientific and ethical discussion on the bioprediction and bioprevention of crime.

We argue here that much of this literature focuses to an unjustifiable degree on crimes that are physical, violent, and/or sexual in nature, while giving little attention to less conventional crimes such as economic and environmental offences. Scientists, and in turn ethicists, tend to focus almost exclusively on biomarkers associated with aggression and low self-control, which are linked with crimes such as murder, assault, and other street crimes often called conventional or blue-collar crimes. In contrast, little scientific or ethical research has been undertaken into biomarkers associated with “white-collar” crimes such as such as fraud or illegal dumping, even though, we will argue, the prospect of biopredicting and biopreventing these crimes has no lesser claim to attention.

We begin by describing the focus of existing literature on biomarkers associated with conventional or blue-collar crime, and explaining why such biomarkers may not be predictive of white-collar crime—white-collar criminals may even, we suggest, tend to exhibit the opposite risk indicators. We then argue that, notwithstanding the reasons for seeking to predict and prevent blue-collar crimes, we have more significant reasons, or at least equally significant ones, for predicting and preventing white-collar crimes. This is because white-collar crime is likely far more costly than blue-collar crime in terms of economic costs, negative health effects, and lives lost—on some accounts, it is 50 times more costly (Friedrichs 2009) and 13 times more deadly (Lynch and Michalowski 2006).

We further argue that the current focus on blue-collar crime may lead to the adoption of measures that are unfair, since those with risk factors for blue-collar crime are more likely to be stigmatised or subjected to intrusive measures than those with risk factors for white-collar crime. This unfairness is exacerbated by the fact that some risk factors for blue-collar crime are also indicators of socio-economic disadvantage, which is itself plausibly unfair. Focusing on biopredicting and biopreventing blue-collar crime may thus tend to compound pre-existing unfairness. We conclude by suggesting that biomarkers for certain psychopathic traits, which appear to be over-represented in senior corporate positions, is one avenue through which biopredictive research can be broadened to include white-collar crime.

Before proceeding, we offer two comments for clarity. The first relates to our focus on *bio*prediction and *bio*prevention of crime, as opposed to, say, the use of demographic or other ecological risk indicators to predict and/or prevent crime. The goals of predicting and preventing crime are certainly best served by using a variety of means, including and especially better understanding the socio-political forces that give rise to it. We do not mean to deny this or to suggest that a biological approach is especially promising—or even that it is likely to be effective if deployed on its own.

However, it is worth emphasising that the brain, and neural activity more generally, mediates biological, psychological, social, and other environmental influences on behaviour. Thus, even if the root causes of crime are primarily psychological, social, and environmental, biomarkers may be useful as a way of identifying the neural states and processes that mediate those causal influences, and biological interventions may be effective in blocking them. Ultimately, however, we focus our analysis on the prospect of using biological predictors and interventions as part of crime prevention efforts because, given that the debate on such applications remains in its nascent stages, there is a strong potential for anticipatory ethical engagement to shape the policies regulating its use. We remain open to the possibility that our conclusions regarding bioprediction and bioprevention could be generalised to crime prediction and crime prevention via other means, but we will not defend that extension to our argument here.

The second comment relates to the distinction between blue-collar and white-collar crime. The terms “blue-collar” and “white-collar” are most commonly used to pick out socio-economic classes, with “blue-collar” deriving from the blue shirts often worn by manual labours in the US in the early twentieth century. As labels for crimes, these terms have historically been used to refer to the crimes associated with these social classes (Sutherland 1940). Hence, blue-collar crimes were the street or conventional crimes associated with lower classes, and white-collar crimes were those associated with elite members of society, and included a broad range of illegal or demonstrably harmful activates that involve the violation of a private or public trust (Friedrichs 2009): fraud, identity theft, money laundering, bribery, insider trading, and so on.

We take the defining feature of white-collar crimes to be that they are illegal, harmful activities that are *not* characterised by direct, intentional violence or direct, intentional invasion of a person’s immediate physical environment. By contrast, we take blue-collar crimes to be illegal, harmful activities that *are* characterised by direct, intentional violence or direct, intentional invasion of a person’s immediate physical environment.^1^

It is important to note that though this distinction is informed by historical class associations, it picks out *types* of crime rather than the social class of the individuals who commit them. A blue-collar crime could be committed by a middle- or upper-class individual, and a white-collar crime by a working class one.

## The Existing Focus on Blue-Collar Crime

The literature on the bioprediction of crime mirrors the popular tendency to associate criminality with inner city residents, minorities, young men, and, crucially, conventionally illegal activities such as homicide, robbery, and sexual assault. Hence, the scientific search for biomarkers is often focused on markers for dispositions such as impulsive aggression and violent behaviour (e.g., Virkkunen et al. 1989; Caspi et al. 2002; Buckholtz and Meyer-Lindenberg 2008) and paraphilia (e.g., Mendez et al. 2000; Mendez and Shapira 2011). The ethical literature follows suit (Beecher-Monas and Garcia-Rill 2006; Berk 2009). Indeed, in the volume *Bioprediction, Biomarkers, and Bad Behavior* (Singh et al. 2013), every chapter that directly deals with the question of biopredicting behaviours associated with criminal activities focuses on physically aggressive, violent, or anti-social behaviour often associated with young men and minorities.

Similarly, in the literature on the bioprevention of crime, the focus is typically on existing or potential treatments for sex offenders, violent individuals, and drug users (e.g., Siever 2008; Douglas 2014; Riva et al. 2015; Chew et al. 2018). In contrast, few if any theorists have looked at biological factors that may dispose individuals to insider trading, diluting medicines or food products for profit, tax evasion, or bribery. As we will argue, such crimes cost much money and many lives.

Of course, the biomarkers associated with blue-collar crime could be the same as those associated with white-collar crime. Thus, even if the explicit focus has chiefly been on blue-collar crime, existing analysis may also, perhaps unintentionally, bear on the prediction of white-collar crime. An influential theory of crime, Gottfredson and Hirschi’s (1990) general theory of crime, argues that the central element behind *all* crime is low self-control. Gottfredson and Hirschi posit that “people who lack self-control will tend to be impulsive, insensitive, physical (as opposed to mental), risk taking, short-sighted, and nonverbal, and they will tend, therefore, to engage in crime and analogous acts.” Moreover, they argue that these dispositions are easily identifiable amongst white-collar criminals: “They too are people of low self-control, people inclined to follow momentary impulse without consideration of the long-term costs of such behavior” (pp. 190–191).

If true, then the focus on biomarkers for impulsivity and aggression could indeed play a role in predicting and preventing both blue and white-collar crime. However, while one of the few meta-analyses evaluating the empirical status of this low self-control theory of crime concluded that the evidence is broadly consistent with it (Pratt and Cullen 2000), not a single study reviewed in it examined corporate offending and other forms of white-collar crime. One study that sought to remedy this deficiency found that corporate offending propensity is unrelated to indicators of low self-control (Simpson and Piquero 2002). Another study, while not looking directly at self-control, found that white-collar criminals have information-processing and other brain superiorities that give them an advantage at perpetrating criminal offences in occupational settings compared to control offenders (Raine et al. 2012). This finding fits well with the notion that white-collar criminals, especially those involved in highly complex, long-running schemes, engage in careful and rational calculation, as opposed to impulsive, short-sighted actions. Crucially, white-collar offenders tend to score higher on some measures of psychopathy than blue-collar offenders (Ragatz et al. 2012)—a point to which we will return below.

Nevertheless, there may be good reasons for the current focus on biopredicting and biopreventing blue-collar crime. First, as individuals, we tend to be most concerned about encountering other individuals prone to directly violent or unwanted sex acts—few would prefer to live near a murderer than an embezzler. Thus, perhaps the focus on blue-collar crime is an appropriate response to what people care about.

Second, the search for biomarkers associated with blue-collar crime has already met with some success: for instance, a sub-type of a gene called MAOA is linked to risk-taking and aggressiveness, at least when combined with maltreatment in childhood (Caspi et al. 2002; Buckholtz and Meyer-Lindenberg 2008). Numerous biomarkers are also implicated in individuals with low self-control (Casey et al. 2011; Tabibnia et al. 2011; Berman et al. 2013), which itself is a strong and consistent predictor of conventional crimes (Gottfredson and Hirschi 1990; Pratt and Cullen 2000; Vazsonyi and Crosswhite 2004; Benda 2005; Chapple 2005). Similarly, several biological interventions may help modulate these types of dispositions. Possibilities include augmenting serotonin levels to reduce aggressive behaviour (Berman et al. 2009), and using non-invasive electric brain stimulation to increase self-control (Lapenta et al. 2014; Loftus et al. 2015), anti-libidinal pharmaceutical agents on sex offenders (Rösler and Witztum 1998), and other pharmacological treatments for substance abusers (Johnson et al. 2007; Kinlock et al. 2008). In contrast, biomarkers and biopreventers for white-collar crimes may simply not exist or may be much harder to reliably identify: the motives behind such crimes may be far more diverse and context-dependent, and biological factors may play a less important role. In other words, researchers interested in bioprediction might be neglecting white-collar crimes because they believe, perhaps with good reason, that it is unlikely they will become biologically predictable or preventable any time soon.

Third, the prevalence and costs of white-collar crimes are notoriously difficult to calculate, with the identities of victims and perpetrators often difficult to ascertain. While a robbery, for instance, usually has a clear victim and perpetrator, with the cost measurable in a relatively straightforward manner, it is typically less clear who suffers when a business shifts numbers on a spreadsheet. Similarly, in corporations with complex bureaucracies and thousands of employees and advisors, it can be difficult to identify the perpetrator(s) when a law is violated. The harms caused by white-collar crimes can also be difficult to quantify and compare. While the harm of white-collar crime can be significant taken as whole, it can also be spread amongst a large number of people, with each only receiving a small fraction of the overall harm. Some might hold that such small harms can never aggregate so as to be worse than a single serious harm suffered by one person (e.g., Kamm 2008). Perhaps uncertainty regarding the costs of white-collar crime and the dispersion of these costs across a large number of people give us reason to focus efforts on blue-collar crime.

We acknowledge, then, that there are reasons supporting the current focus on blue-collar crimes in the literature on bioprediction and bioprevention. However, in the following sections, we argue that there are also powerful reasons on the other side of the ledger. The first of these pertains to the costs of the two types of crime: though we do not know what the full costs of white-collar crime are, from what little data is available, even conservative estimates suggest they are far larger than those associated with blue-collar crime.

## The Deadly Costs of White-Collar Crime

Examples of white-collar crime abound: pharmacists dilute cancer drugs to boost their profits (Draper 2003), car companies cheat emissions tests (Schiermeier 2015), drug companies suppress worrying findings about their products (Gøtzsche 2012), corporations illegally dump poisonous toxins that kill thousands (Mokhiber 1988), producers of agricultural feed slip prohibited ingredients into their products (Spiegel 2011), and billions of dollars are embezzled by kleptocratic regimes around the world (Transparency International 2004).

As mentioned, ascertaining the full impact of white-collar crime raises unique epistemic challenges. Some indicative figures can nevertheless help contrast its costs with those of blue-collar crime. In the UK, for instance, while violent crimes cost an estimated £124 billion a year, including the cost of police investigations, courts and prison expenditures and loss in productivity (IEP 2013), fraud on its own is estimated to cost £190 billion annually (Crowe UK 2017). In the US, losses from some forms of white-collar crime were in the range of $250 billion in a year, compared to $4 billion from conventional offences, such as burglary and robbery (Friedrichs 2009). On some measures, losses to white-collar crime in the US have been estimated to be as high as $1 trillion annually (Schlegel 2000; Ragatz et al. 2012). These are vastly different estimates, and it is clear that there is no uniform definition or metric of the costs of white-collar crime in use here. Nevertheless, for most scholars of this topic, the question is typically whether white-collar crime is 50 or merely 10 times more costly than blue-collar crime (Friedrichs 2009).

Financial cost aside, white-collar crime is often also deadly. Recall that the harms of these types of offences are often diffused amongst thousands of only slightly harmed or wronged individuals. However, this is often not the case, and historic and more recent cases can serve to illustrate the fatalities directly arising from them. Consider, for instance, the Bhopal disaster in India: a pesticide plant had a massive gas leak in December 1984 that led to some 8000 deaths and half a million injuries (Eckerman 2005). In Japan, in the 1950 s, a petrochemical corporation dumped enormous amounts of toxic waste into the sea, leading to residents of a nearby village, Minamata, developing severe brain, and body dysfunctions, including birth defects and blindness (Mokhiber 1988). In both cases, and there are many such cases (Mokhiber 1988), subsequent investigations showed that the corporations involved had been knowingly negligent and cut corners on safety.

On an industry-wide scale, consider the case of asbestos, a group of fibrous, heat-resistant minerals that was once marketed as the “magic mineral.” In 2004 alone, the World Health Organization (WHO) estimated that it led to 107,000 deaths and 1.5 million Disability Adjusted Life Years due to lung cancer, mesothelioma, and asbestosis (WHO 2015). Malignant mesothelioma is thought to be almost entirely attributable to asbestos exposure and is almost universally lethal. Of course, not all of this mortality is attributable to criminal activity, and criminal convictions for asbestos-related offences have been notoriously difficult to obtain. However, the health risks of asbestos exposure were apparent to industry insiders and regulators as early as the 1930s (Merewether and Price 1930), and its cancer risks well established by the 1960s (Smith 2005), yet significant exposure continued until the 1980s in most developed countries and continues today in much of the developing world (Røe and Stella 2015). There is evidence that criminal negligence and other criminal activities have contributed substantially to asbestos exposures and thus to asbestos-related deaths (e.g., United States vs. Thorn 2013; McCulloch and Tweedale 2008; Reuters 2013).

For a more recent example, consider the so-called “diesel dupe.” In 2015, car maker Volkswagen was found to have used software in 11 million of its cars that detects when they are being tested, changing their engine performance in order to improve their emission test results. On the road, however, their engines emitted nitrogen oxide at up to 40 times the maximum allowed level in the US (Hotten 2015). A growing list of car manufacturers has since been found to cheat regulatory tests (Carrington 2015). Meanwhile, the most comprehensive global analysis on air pollution and health, commissioned by *The Lancet*, linked air pollution to 9 million deaths worldwide every year, costing in the trillions of dollars (Landrigan et al. 2017). Even if illegal car emissions contribute to a small fraction of these deaths and costs, this would still be a substantial figure.

But perhaps the most devastating form of white-collar crime is political corruption, which we define here as the illegal abuse of public office for private gain. Corruption is one of the most reliable ways to stifle economic growth (Wei 2000; Mo 2001; Drury et al. 2006). But corruption also kills: one study estimates that the deaths of 140,000 children are attributable to corruption each year (Hanf et al. 2011). It can be pernicious in other ways too: 83% of all deaths from building collapses due to earthquakes over the past 30 years occurred in particularly corrupt countries, where bribes are regularly used to bypass inspection and licensing processes, as well as to reduce construction costs and thereby compromise building safety (Ambraseys and Bilham 2011).

Businesses and individuals pay an estimated $1.5 trillion in bribes each year, or 2% of global GDP (World Bank 2017). Aside from evading building inspection, these can be used to influence governments’ choices of firms to supply goods, services, and works; to influence the allocation of government benefits like subsidies for enterprises or access to pensions; to lower taxes; and to influence legal outcomes by, for instance, preventing the prosecution of illegal polluting (World Bank 1997). As such, public spending on key infrastructures such as health and education is lower in corrupt states because funds are diverted by corrupt officials to projects that are more difficult to monitor and, therefore, easier to embezzle from (Mauro 1998).

According to the anti-poverty organisation, One, the world’s developing countries are deprived of at least $1 trillion each year due to money laundering, tax evasion, and embezzlement (One 2014). This money could be used to avert 3.6 million people dying each year between 2015 and 2025 in low-income countries. Arguably, therefore, the greatest problem facing the developing world is not famine or pestilence, but corruption.

To take a concrete example, consider a small yet illustrative case in Bangladesh where, as part of a plea entered with the US government, Siemens Bangladesh admitted that between May 2001 and August 2006 it paid $5.3 million to various Bangladeshi officials in return for preferential consideration on public contracts (United States v. Siemens Bangladesh 2008). One of those officials, Arafat “Koko” Rahman, reportedly sent $3 million of those bribes to bank accounts in Singapore. While this amount is pocket change in contrast to the estimated $1 trillion lost to corruption each year, if returned to Bangladesh, Transparency International (2015) estimates it would be enough to pay for 55,414 tuberculosis treatments, for over 300,000 children to attend school for a year, or to build 1147 flats for slum dwellers.

Ultimately, corruption erodes confidence in state institutions and the rule of law as mechanisms for conflict resolution, which can encourage citizens to resort to violence as an alternative. More generally, white-collar crime increases feelings of alienation and cynicism in a society, which in themselves contribute to more conventional crimes (Friedrichs 2009). White-collar crime, and especially corruption in developing countries, is an important contributor to poverty and inequality (Gupta et al. 2002; Gyimah-Brempong 2002). Yet poverty and inequality—especially when inequality is visible through conspicuous consumption—play significant roles in the causation of violent crime (Hicks and Hicks 2014). To that extent, the cost of white-collar crime also extends to its contribution to creating the circumstances in which conventional crimes are more likely to take place. For this reason alone, the continued neglect of white-collar crime in the associated bioprediction literature seems unjustified. The massive overall financial and human costs of white-collar crime strengthen the case for that conclusion.

## Biomarkers, Inefficiency, and Unfairness

Besides the lack of any positive justification, there is further reason that neglecting white-collar crime is problematic. One of the issues arising from the scientific focus on, and seeming availability of, biomarkers associated with violent behaviours is that it may result in criminal justice systems prioritising blue-collar crimes in their efforts at prediction and prevention (Wolpe 2013). This is not to claim that the ethical discussion concerning the bioprediction and bioprevention of blue-collar crime will lead to a greater investment of resources in this area; after all, much of this ethical discussion is critical of bioprediction and bioprevention. Rather, it is the scientific research, and the technological developments that it enables, that may contribute to that outcome. This is problematic to the extent that those resources would come at the expense of further neglecting white-collar crime, which may not be associated with these markers. To the extent that white-collar crime is more costly than blue-collar crime, and in particular to the extent that white-collar crime can foster in a society the conditions conducive to blue-collar crime, then this is an inefficient use of resources.

Moreover, the focus on blue-collar crimes might also contribute to unfairness. The greater application of crime prevention measures to those with risk factors for blue- rather than white-collar crime might be intrinsically unfair. It may also produce or exacerbate pre-existing unfair inequality to the extent that individuals who commit blue-collar crimes such as criminally aggressive acts tend to already be disadvantaged, whereas those who commit white-collar crimes are not.

For instance, poverty is consistently found to be a driver for violent offending amongst young people (Ludwig et al. 2001; McAra and McVie 2016). Even if poverty is not directly *causing* blue-collar crimes, poverty and inequality are clearly associated with blue-collar crime (Blau and Blau 1982; Patterson 1991; Hsieh and Pugh 1993). Homelessness is also over-represented in prison populations, with 15% of new convicts in the UK reportedly being homeless before incarceration (Ministry of Justice 2012). To that extent, people who commit many of the crimes on which this literature focuses are born into or live in poverty or similarly disadvantageous socio-economic states. Mental health disorders are also disproportionally present amongst convicts (Light et al. 2013), with some 20–30% of all UK inmates suffering from learning disabilities (Loucks 2007). Given that the vast majority of inmates are incarcerated for blue-collar crimes, we can infer that many individuals who commit such crimes already suffer from socio-economic disadvantage and disorders that already limit life opportunities in some way. Furthermore, disadvantaged ethnic groups with a history of discrimination also tend to be over-represented in prisons (e.g., Department of Corrections 2007; Minton and Zeng 2015). Hence, the use of biomarkers to predict the likelihood of such typically disadvantaged individuals committing crimes may compound their disadvantage by introducing intrusive measuring or stigmatising labels or disadvantage-exacerbating forms of interventions, such as incarceration.

The use of labels here may also be directly harmful to innocent individuals and counter-productive to reducing crime. There is evidence that the mere label of “felon” can double the rate of recidivism compared to those who are equally guilty of past criminal acts but who have had the label of “felon” withheld (Chiricos et al. 2007). This gives us reason to be wary of labels that suggest people who are innocent are *potential* felons, especially if that potentiality resides in their biology. A biological label that denotes a disposition toward criminality may impede the likelihood of rehabilitation. The view that such dispositions are innate may interfere with one’s likelihood of outgrowing or changing those dispositions.

This may be particularly significant in the case of children. Compelling evidence suggests that labelling highly influences the scholastic career and future employment of children (Sampson and Laub 1997). This impact may be heightened in the case of biological labelling, such as having a “low IQ”—such a label impacts not only children’s academic motivation and scholastic achievement, but also how they are treated by others (Sampson and Laub 1997). A label for being biologically predisposed toward bad behaviour can further exacerbate a propensity toward bad behaviour, both because of the child’s self-perception, and because adults may be less inclined to believe the child is merely going through a developmental phase, and hence may be more likely to give up on helping the child. In that way, labels associated with biomarkers may act to stigmatise and increase the likelihood of committing crimes by individuals who are already disadvantaged in life, both socio-economically and, according to these biomarkers, biologically.

In contrast to this, biomarkers associated with white-collar crime are less likely to raise these particular issues. If white-collar crimes are typically perpetrated by individuals in legitimate and often respectable occupations, concerns about compounding disadvantage do not arise as strongly, if at all. Hence, the focus on blue-collar crime here is likely to have a greater unfairness-promoting effect than would a more balanced focus on both types of crime.

## Biomarkers for White-Collar Crime?

In making the above arguments, a crucial question that arises is whether there are identifiable and reliable biomarkers associated with white-collar crime. On the one hand, it might seem that any biological associations with white-collar crime would be weak and highly complex, particularly given that many such crimes are committed by institutions—whether corporate or political—or by groups of people working together, or at least colluding, rather than individual people. White-collar crime of an institutional or collective variety is likely to be far more strongly associated with societal factors—such as incentive structures and bureaucratic processes—than with the biological characteristics of those working within the institutions and collectives.

We will not pursue institutional or collective misbehaviour further here. Rather, we will address cases where the characteristics—including biological characteristics—and behaviours of individuals can have significant effects, for example, where those individuals hold senior positions in influential organisations. We will focus particularly on cases where such individuals exhibit what is variously known as “corporate psychopathy,” “successful psychopathy” (Board and Fritzon 2005), or “executive psychopathy” (Morse 2004). These individuals possess a cluster of personality traits that, we will argue, could dispose them to perpetrate white-collar crimes, and to encourage others without those traits to also commit these crimes. We propose that investigating whether such personality traits correlate with certain biological characteristics may be one way to extend bioprediction, and perhaps even bioprevention, to white-collar crime.^2^

A corporate psychopath is not a psychopath in the clinical sense of this term. Clinical psychopathy is characterised by four features: callous affect, interpersonal manipulation, erratic lifestyle, and anti-social behaviour (Williams et al. 2007). The first two features manifest in the absence of feelings like empathy, guilt, and remorse when others are manipulated to further the psychopath’s highly egocentric interests. The latter two features are associated with a propensity toward impulsivity and irresponsibility, often resulting in violent, anti-social outbursts (Hare and Neumann 2008, 2009). To be diagnosed as a psychopath is to be deemed to possess these four traits to a high degree over time.

What is of interest to us here is that some individuals may score highly in callous affect and interpersonal manipulation, but not in erratic lifestyle and anti-social tendencies (Babiak and Hare 2007; Bate et al. 2014; Lingnau et al. 2017). Such individuals may be thought of as “successful psychopaths,” or “corporate psychopaths,” as we call them here (Boddy 2011b). Their lifestyles are not necessarily erratic and they are less prone to overt anti-social behaviours, but they lack empathy and are able to manipulate others through superficial charm and other means without remorse to further their own long-term interests (Boddy 2011c).

These characteristics are often highly regarded and conducive to success in the domains of business and politics. Egocentricity can be perceived as reflecting a high degree of self-confidence, and even the lack of any discernible long-term goals might be praised as “visioning” (Babiak 2006). As such, it is no surprise that these traits are over-represented in leaders of large, powerful organisations. Some evidence suggests corporate psychopaths constitute 3–6% of business managers (Babiak et al. 2010), and more than 20% of supply chain managers (Fritzon et al. 2017). One study took three groups—senior business managers, psychiatric patients, and hospitalised criminals—and profiled them for a number of personality traits (Board and Fritzon 2005). The studies found that certain traits associated with psychopathy were in some cases *more* common among business managers than in clinically psychopathic patients—specifically traits such as charm, egocentricity, lack of empathy, and persuasiveness. The main difference between the groups was that the senior business mangers did not exhibit the erratic and anti-social tendencies associated with secondary psychopathy—that is, they were less likely to be physically aggressive, impulsive, and hence less likely to commit blue-collar crimes.

Corporate psychopaths as such may be less likely than other corporate workers to have qualms about engaging in white-collar crime if it will further their interests. For instance, Lingnau et al. (2017) found a highly significant relationship between personality traits associated with corporate psychopathy and the acceptance of accounting fraud and insider trading. Some have even argued that corporate psychopaths have played central roles in major corporate scandals in recent years, such as the Enron scandal in 2001, and the Chinese milk scandal in 2008. In the latter case, it transpired that top managers of the dairy company Sanlu were fully aware that a toxic compound used in making plastics was being added to its milk products, and which led to the poisoning of 300,000 people, mostly infants (Xiu and Klein 2010; Lingnau and Dehne-Niemann 2015). Others have gone further, suggesting that corporate psychopaths played crucial roles in the 2007 global financial crisis. Specifically, Boddy (2011a) suggests a “corporate psychopaths theory of the global financial crisis,” which highlights how changes in the way people are employed at corporations over the past four decades have made it easier for corporate psychopaths to rise to senior positions, and how from there they used their personal greed to create the crisis.

For our purposes, we remain agnostic about whether such individuals played any significant roles in large economic phenomena such as global financial crises. Regardless, it is certainly plausible that corporate psychopaths in senior positions, with their abilities to persuade and manipulate others, can influence the moral climate of entire organisations—that is, alter the scope of what are perceived as acceptable norms and behaviours. This might in turn normalise the sorts of behaviours that, en masse, lead to institutionalising acts of white-collar crime (Ashforth and Anand 2003; Boddy 2011c). The same could be argued for political organisations, although this has received far less, if any, attention. Political psychopaths may do as much if not more harm than corporate psychopaths, especially in countries where there are fewer checks and balances on the conduct of politicians.

We similarly remain agnostic as to whether this construct of corporate psychopathy ought to be treated as a genuine personality disorder for clinical purposes. More important in this context is whether traits such as callous affect and a heightened disposition to manipulate others for one’s own ends might be reliably detectable, thereby enabling organisations to better screen for individuals who possess these traits to a high degree.

One way to do that would be to deploy measures of psychopathy when hiring individuals for senior positions at highly influential organisations. In some countries, such measures are already in use when hiring for positions of critical public safety, such as police, fire, and nuclear power plant operators (Lowman 1989). Individuals at the highest levels of large political and corporate organisations are, like nuclear power plant operators, in positions that can inflict massive harm. Regulations that would require prospective candidates for such high-level jobs to undergo tests for psychopathy, amongst other tests of mental health, are arguably long overdue (e.g., Lee and Eisen 2018).

One limitation of such a proposal, however, is that individuals such as corporate psychopaths appear particularly skilled at masking some of their traits by manipulating tests that are designed to reveal those very traits (Bate et al. 2014). That is, we have reasons to be sceptical that an interview process, clinically structured or not, can reliably detect corporate psychopaths: the higher intelligence and increased self-control mean they may be more able to evade behavioural or psychological testing.

This makes the possibility of identifying reliable biomarkers associated with such personality traits a particularly promising domain of research for corporate psychopaths. It offers the potential of screening for such individuals regardless of how clever they might be at manipulating self-report-based tests.

As things stand, there is some evidence that brain imaging techniques can identify specific abnormalities in the brains of psychopaths (Harenski et al. 2010; Ermer et al. 2012). For instance, Hosking et al. (2017) used a mobile MRI scanner to image the brains of prison inmates and found that the brains of people who score highly on measures of psychopathy, with all four factors exhibited, have significantly weaker connections between a region in the brain associated with evaluating a reward (the nucleus accumbens) and a region associated with decision making (the ventromedial prefrontal cortex). This weakened connection is taken to partly explain psychopaths’ tendencies to overvalue immediate rewards and neglect the consequences of imprudent or immoral behaviour. Another study using MRI scans found that individuals with psychopathic tendencies have reduced grey matter in regions of the brain associated with understanding other people’s emotions and intentions (Gregory et al. 2012). There is also a growing list of specific genetic variations associated with psychopathic tendencies (Viding et al. 2010).

Despite identifying these potential biomarkers, it remains unclear how generalisable they are and how specific they might be to clinical psychopathy (Hardcastle 2013), let alone how predictive they might be of corporate psychopathy. Little if any research has been conducted here, one reason being due to the relative lack of interest in white-collar crimes in bioprediction research.

Nevertheless, the specific personality traits of this construct, such as callous affect and extreme egocentricity, may correlate with certain biological characteristics. For instance, callous unemotional traits appear highly heritable and stable throughout at least childhood and adolescence: a genetic understanding of callous-unemotional traits is a growing area of research that may yield reliable biomarkers (Viding and McCrory 2013). Similarly, there has been some research linking hormonal correlates with extreme narcissism—in particular, higher cortisol levels seem at least marginally related with an unhealthy degree of narcissism (Reinhard et al. 2012). As these biomarkers become better understood, particular combinations of them in individuals may be found to correlate with corporate psychopathy.

We do not wish to offer a fully developed proposal for how biomarkers for corporate psychopathy ought to be used, should they be discovered. However, we do wish to offer some preliminary suggestions by way of responding to some ethical concerns that might be thought to rule out their permissible use.

First, we suggest that biomarkers for corporate psychopathy should generally be used as grounds for *deprioritising* candidates for political or senior corporate positions rather than as a basis for absolutely excluding candidates. This would mitigate the worry that such biomarkers might be used in a way that would exclude individuals who in fact have other, compensating qualities.

Second, we propose that bioprediction should take into account the widest available range of biomarkers, including markers for desirable traits that could offset undesirable ones. Simply focusing on a narrow set of biomarkers may exclude people who could make valuable contributions. And, of course, any such biopredictive exercise would need to be conducted in concert with a host of other non-biological predictive and evaluative factors, such as one’s professional track record, and so on.

Third, we would not favour a blanket approach that sought to screen out individuals with corporate psychopathic traits from *all* senior corporate and political positions. Some psychopathic traits may be valuable in the context of certain professions and/or organisations. For instance, the ability to remain unaffected by stressful situations, to charm and persuade others, may be desirable qualities in those who occupy some roles (Dutton 2013).

Fourth, we suggest that, where feasible, biomarkers for corporate psychopathy should be used not to exclude certain people from professions, but to identify those who would warrant enhanced surveillance of their decisions and behaviour (Baum and Savulescu 2013). This would lead to some stigmatisation, but not exclusion from that particular job or profession. The charming and persuasive character with some psychopathic traits would be subjected to greater oversight and monitoring. Greater oversight and co-ordination is arguably in any case warranted in the corporate and political world.

As pointed out in the previous section, using biomarkers to predict white-collar crime—and in this case specifically to screen individuals before awarding them highly influential roles in corporate or political organisations—does not raise the same ethical concerns associated with using biomarkers to predict blue-collar crime. Blue-collar criminals are often already disadvantaged, and identifying them with a biomarker that is predictive of further crime may exacerbate that disadvantage. In contrast, individuals vying for CEO or high-level political positions are typically not disadvantaged in that way. Missing out on such an opportunity due to biomarkers associated with destructive personality traits would not entail compounding disadvantage as it often does with blue-collar offenders. Of course, steps could be taken to reduce the chances of stigmatising such candidates, such as by ensuring confidentiality regarding the specific reasons they failed to attain certain positions. And at-risk individuals could be subjected to greater oversight and monitoring. However, the key point here is that, given the high stakes and the relatively small number of individuals who are at the top of such organisations, the use of biomarkers in this case would pose fewer concerns about injustice compared to its use in preventing blue-collar crime.

One final point. Of course, false positives are always a concern with such tests. This refers to the possibility of a test suggesting a candidate is likely a corporate psychopath, even though he or she is not. No doubt, the use of any such test would only be justified if its incidence of false positives was quite small. However, it is worth conceding that, as with other crime prevention efforts, some degree of false positives is inevitable.

## Conclusion

The research on biopredicting and biopreventing crime tends to neglect white-collar crime, despite its likely far greater cost when compared to the conventional offences associated with blue-collar crime. This approach may not only lead to an inefficient approach to reducing the overall costs of crime; it may also lead to unfair outcomes, further burdening individuals with risk indicators that tend to correspond with socio-economic disadvantage.

It is not immediately clear how this research on bioprediction and bioprevention can be extended to include white-collar crime. Nevertheless, we have argued that some of the traits associated with personalities that are characteristic of corporate psychopathy, such as callous affect and interpersonal manipulation, are a promising place to begin the search for biomarkers associated with at least some forms of white-collar crime. Moreover, corporate psychopaths are more likely to escape detection by behavioural and psychological testing, making the search for biomarkers more important. Future research ought to investigate such potential biorisk indicators. If reliable biomarkers are successfully identified, this would bring us one step closer to developing screening measures that could be used to inform recruitment decisions for high-level corporate and political positions, or to trigger greater oversight and monitoring of the decisions and behaviour of those identified as being at risk.

## Footnotes

1 There may, of course, be exceptions to this distinction. Consider thieving, which is typically considered a blue-collar crime, and cybercrime, which is typically considered a white-collar crime. It is not clear there will be a relevantly different “brain picture” between offenders of these two crimes. However, we do not claim that all white-collar criminals can be distinguished by a unified set of traits that could entail a single biopredictive or biopreventive strategy for all white-collar crime. Different types of white-collar crime may require different approaches. We thank an anonymous reviewer for highlighting this point.

2 To be clear: this is not a proposal for preventing white-collar crime *in general*—no doubt any such preventative efforts will require a host of institutional and perhaps even cultural changes related to how we incentivise employees and managers. Instead, this is merely an illustration of one way bioprediction might be deployed in this context.

## References

[CR1] Ambraseys N, Bilham R (2011). Corruption Kills. Nature.

[CR2] Ashforth B, Anand V (2003). The Normalization of Corruption in Organization. Research in Organizational Behavior.

[CR3] Babiak, P. 2006. “From Darkness into the Light: Psychopathy in Industrial and Organizational Psychology.” In *The Psychopath: Theory, Research, and Practice*, edited by Hervé, H. and Yuille, J. Routledge: 411–428.

[CR4] Babiak P, Hare R (2007). Snakes in Suits: When Psychopaths Go to Work.

[CR5] Babiak P, Neumann C, Hare T (2010). Corporate Psychopathy: Talking the Walk. Behavioral Sciences & the Law.

[CR6] Bate C, Boduszek D, Dhingra K (2014). Psychopathy, Intelligence and Emotional Responding in a Non-Forensic Sample: An Experimental Investigation. Journal of Forensic Psychiatry & Psychology.

[CR7] Baum M, Savulescu J, Singh I, Sinott-Armstrong W, Savulescu J (2013). Behavioural Biomarkers: What Are They Good For? Towards the Ethical Use of Biomarkers. Bioprediction, Biomarkers, and Bad Behaviour: Scientific, Legal and Ethical Challenges.

[CR8] Beecher-Monas E, Garcia-Rill E (2006). Genetic Predictions of Future Dangerousness: Is There a Blueprint for Violence?. Law and Contemporary Problems.

[CR9] Benda B (2005). The Robustness of Self-Control in Relation to Form of Delinquency. Youth & Society.

[CR10] Berk R (2009). The Role of Race in Forecasts of Violent Crime. Race and Social Problems.

[CR11] Berman M, Yourganov G, Askren M (2013). Dimensionality of Brain Networks Linked to Life-Long Individual Differences in Self-Control. Nature Communications.

[CR12] Berman M, McCloskey M, Fanning J (2009). Serotonin Augmentation Reduces Response to Attack in Aggressive Individuals. Psychological Science.

[CR13] Blau J, Blau P (1982). The Cost of Inequality: Metropolitan Structure and Violent Crime. American Sociological Review.

[CR14] Board B, Fritzon K (2005). Disordered Personalities at Work. Psychology, Crime & Law.

[CR15] Boddy C (2011). The Corporate Psychopaths Theory of the Global Financial Crisis. Journal of Business Ethics.

[CR16] Boddy C (2011). Corporate Psychopaths: Organizational Destroyers.

[CR17] Boddy C (2011). Corporate Psychopaths, Bullying and Unfair Supervision in the Workplace. Journal of Business Ethics.

[CR18] Buckholtz J, Meyer-Lindenberg A (2008). MAOA and the Neurogenetic Architecture of Human Aggression. Trends in Neurosciences.

[CR19] Carrington, D. 2015. “Four More Carmakers Join Diesel Emissions Row.” *The Guardian*. Available from: http://www.theguardian.com/environment/2015/oct/09/mercedes-honda-mazda-mitsubishi-diesel-emissions-row. Accessed 14 Apr 2018.

[CR20] Casey B, Somerville L, Gotlib I (2011). Behavioral and Neural Correlates of Delay of Gratification 40 Years Later. Proceedings of the National Academy of Sciences.

[CR21] Caspi A, McClay J, Moffitt T (2002). Role of Genotype in the Cycle of Violence in Maltreated Children. Science.

[CR22] Chapple C (2005). Self-control, Peer Relations, and Delinquency. Justice Quarterly.

[CR23] Chew C, Douglas T, Faber N, Birks D, Douglas T (2018). Biological Interventions for Crime Prevention. Treatment for Crime.

[CR24] Chiricos T, Barrick K, Bales W (2007). The Labeling of Convicted Felons and Its Consequences for Recidivism. Criminology.

[CR25] Crowe UK. 2017. “Annual Fraud Indicator 2017 Identifying the Cost of Fraud to the UK Economy.” Available from: https://www.crowe.com/uk/croweuk/news/annual-fraud-indicator-2017. Accessed 2 May 2018.

[CR26] Department of Corrections (2007). “Over-Representation of Maori in the Criminal Justice System.” Available from: http://www.corrections.govt.nz/resources/research_and_statistics/over-representation-of-maori-in-the-criminal-justice-system.html. Accessed 22 July 2018.

[CR27] Douglas T (2014). Criminal Rehabilitation Through Medical Intervention: Moral Liability and the Right to Bodily Integrity. Journal of Ethics.

[CR28] Draper, R. 2003. “The Toxic Pharmacist.” *The New York Times*. Available from: https://www.nytimes.com/2003/06/08/magazine/the-toxic-pharmacist.html. Accessed 23 Apr 2018.

[CR29] Drury C, Krieckhaus J, Lusztig M (2006). Corruption, Democracy, and Economic Growth. International Political Science Review.

[CR30] Dutton K (2013). The Wisdom of Psychopaths.

[CR31] Eckerman I (2005). The Bhopal Saga: Causes and Consequences of the World’s Largest Industrial Disaster.

[CR32] Ermer E, Cope L, Calhoun V (2012). Aberrant Paralimbic Gray Matter in Criminal Psychopathy. Journal of Abnormal Psychology.

[CR33] Friedrichs D (2009). Trusted Criminals: White Collar Crime In Contemporary Society.

[CR34] Fritzon, K., Bailey, C., Croom, S. et al. 2017. “Problem Personalities in the Workplace: Development of the Corporate Personality Inventory.” In *Psychology and Law in Europe: When West Meets East*, edited by Granhag P., Bull R., Shaboltas A., and Dozortseva E., CRC Press: 139–166.

[CR35] Gottfredson M, Hirschi T (1990). A General Theory of Crime.

[CR36] Gregory S, Ffytche D, Simmons A (2012). The Antisocial Brain: Psychopathy Matters: A Structural MRI Investigation of Antisocial Male Violent Offenders. Archives of General Psychiatry.

[CR37] Gøtzsche P (2012). Big Pharma Often Commits Corporate Crime, and this Must Be Stopped. BMJ.

[CR38] Gupta S, Davoodi H, Alonso-Terme R (2002). Does Corruption Affect Income Inequality and Poverty?. Economics of Governance.

[CR39] Gyimah-Brempong K (2002). Corruption, Economic Growth, and Income Inequality in Africa. Economics of Governance.

[CR40] Hanf M, Van-Melle A, Fraisse F (2011). Corruption Kills: Estimating the Global Impact of Corruption on Children Deaths. PLoS ONE.

[CR41] Hardcastle V (2013). It Isn’t as Simple as It Seems: Understanding and Treating Psychopathy. American Journal of Bioethics Neuroscience.

[CR42] Hare R, Neumann C (2009). Psychopathy: Assessment and Forensic Implications. Canadian Journal of Psychiatry.

[CR43] Hare R, Neumann C (2008). Psychopathy as a Clinical and Empirical Construct. Annual Review of Clinical Psychology.

[CR44] Harenski C, Harenski K, Shane M (2010). Aberrant Neural Processing of Moral Violations in Criminal Psychopaths. Journal of Abnormal Psychology.

[CR45] Hicks D, Hicks J (2014). Jealous of the Joneses: Conspicuous Consumption, Inequality, and Crime. Oxford Economic Papers.

[CR46] Hosking, J. Kastman, E., Dorfman, H. et al. 2017. “Disrupted Prefrontal Regulation of Striatal Subjective Value Signals in Psychopathy.” *Neuron* 95 (1): 221–231.e4. 10.1016/j.neuron.2017.06.030 PMC5796650 28683266

[CR47] Hotten, R. 2015. “Volkswagen: The Scandal Explained.” *BBC News*. Available from: http://www.bbc.co.uk/news/business-34324772. Accessed 22 Apr 2018.

[CR48] Hsieh C-C, Pugh M (1993). Poverty, Income Inequality, and Violent Crime: A Meta-Analysis of Recent Aggregate Data Studies. Criminal Justice Review.

[CR49] IEP. 2013. “UK Peace Index: Exploring the Fabric of Peace in the UK from 2003–2002.” *Institute for Economics and Peace*. Available from: http://economicsandpeace.org/wp-content/uploads/2015/06/UK_Peace_Index_report_2013_0.pdf.

[CR50] Johnson B (2007). Topiramate for Treating Alcohol Dependence: A Randomized Controlled Trial. Journal of the American Medical Association.

[CR51] Kamm, F. 2008. *Intricate Ethics: Rights, Responsibilities, and Permissible Harm*. Oxford Ethics Series. Oxford, New York: Oxford University Press.

[CR52] Kinlock T, Gordon M, Schwartz R (2008). A Study of Methadone Maintenance for Male Prisoners: 3-Month Postrelease Outcomes. Criminal Justice and Behavior.

[CR53] Landrigan P, Fuller R, Acosta N (2017). The *Lancet* Commission on Pollution and Health. The Lancet.

[CR54] Lapenta O, Di Sierve K, Coutinho de Macedo E (2014). Transcranial Direct Current Stimulation Modulates ERP-Indexed Inhibitory Control and Reduces Food Consumption. Appetite.

[CR55] Lee, B., and Eisen, N. 2018. “On Trump’s Mental Fitness, the Experts Are Silenced and the Public’s in the Dark.” *USA Today*. Available from: https://www.usatoday.com/story/opinion/2018/01/19/trumps-mental-fitness-experts-silenced-public-dark-bandy-lee-norman-eisen-column/1043765001/. Accessed 9 Aug 2018.

[CR56] Light, M., Grant, E., and Hopkins K. 2013. “Gender Differences in Substance Misuse and Mental Health Amongst Prisoners. London: Ministry of Justice.” Available from: https://assets.publishing.service.gov.uk/government/uploads/system/uploads/attachment_data/file/220060/gender-substance-misuse-mental-health-prisoners.pdf. Accessed 29 July 2018.

[CR57] Lingnau V, Fuchs F, Dehne-Niemann T (2017). The Influence of Psychopathic Traits on the Acceptance of White-Collar Crime: Do Corporate Psychopaths Cook the Books and Misuse the News?. Journal of Business Economics.

[CR58] Lingnau V, Dehne-Niemann T, Vorbohle K, Quandt J (2015). When Managing Is Damaging – Corporate Psychopaths and Their Potential Impact on Stakeholders’ Achievement of Objectives in the Global Supply Chain. Verantwortung in Der Globalen Wertschöpfung.

[CR59] Loftus A, Yalcin O, Baughman F (2015). The Impact of Transcranial Direct Current Stimulation on Inhibitory Control in Young Adults. Brain and Behavior.

[CR60] Loucks N (2007). No One Knows: Offenders with Learning Difficulties and Learning Disabilities – Review of Prevalence and Associated Needs.

[CR61] Lowman R (1989). Pre-Employment Screen for Psychopathy: A Guide to Professional Practice.

[CR62] Ludwig J, Duncan G, Hirschfield P (2001). Urban Poverty and Juvenile Crime: Evidence from a Randomized Housing-Mobility Experiment. Quarterly Journal of Economics.

[CR63] Lynch M, Michalowski R (2006). Primer in Radical Criminology: Critical Perspectives on Crime, Power, and Identity.

[CR64] Mauro P (1998). Corruption and the Composition of Government Expenditure. Journal of Public Economics.

[CR65] McAra L, McVie S (2016). Understanding Youth Violence: The Mediating Effects of Gender, Poverty and Vulnerability. Journal of Criminal Justice.

[CR66] McCulloch J, Tweedale G (2008). Defending the Indefensible: The Global Asbestos Industry and Its Fight for Survival.

[CR67] Mendez M, Shapira J (2011). Pedophilic Behavior from Brain Disease. Journal of Sexual Medicine.

[CR68] Mendez M, Chow T, Ringman J (2000). Pedophilia and Temporal Lobe Disturbances. Journal of Neuropsychiatry and Clinical Neurosciences.

[CR69] Merewether, E., and Price, C. 1930. “Report on Effects of Asbestos Dust on the Lungs and Dust Suppression in the Asbestos Industry.” London: His Majesty’s Stationary Office. Available from: https://www.jdsupra.com/legalnews/report-on-effects-of-asbestos-dust-on-th-15946/. Accessed 3 May 2018.

[CR70] Ministry of Justice 2012. “Accommodation, Homelessness and Reoffending of Prisoners: Results from the Surveying Prisoner Crime Reduction (SPCR) survey.” London: Ministry of Justice. Available from: https://www.gov.uk/government/publications/accommodation-homelessness-and-reoffending-of-prisoners. Accessed 22 July 2018.

[CR71] Minton, T., and Zeng, Z. 2015. “Jail Inmates at Midyear 2014.” *Bureau of Justice Statistics*. Available from: https://www.bjs.gov/content/pub/pdf/jim14.pdf. Accessed 29 July 2018.

[CR72] Mo P (2001). Corruption and Economic Growth. Journal of Comparative Economics.

[CR73] Mokhiber R (1988). Corporate Crime and Violence: Big Business Power and the Abuse of Public Trust.

[CR74] Morse G (2004). Executive Psychopaths. Harvard Business Review.

[CR75] One. 2014. “Trillion Dollar Scandal.” Available from: https://www.one.org/international/policy/trillion-dollar-scandal/. Accessed 15 June 2018.

[CR76] Patterson E (1991). Poverty, Income Inequality, and Community Crime Rates. Criminology.

[CR77] Pratt T, Cullen F (2000). The Empirical Status of Gottfredson and Hirschi’s General Theory of Crime: A Meta-Analysis. Criminology.

[CR78] Ragatz L, Fremouw W, Baker E (2012). The Psychological Profile of White-Collar Offenders: Demographics, Criminal Thinking, Psychopathic Traits, and Psychopathology. Criminal Justice and Behavior.

[CR79] Raine A, Laufer W, Yang Y (2012). Increased Executive Functioning, Attention, and Cortical Thickness in White-Collar Criminals. Human Brain Mapping.

[CR80] Reinhard D, Konrath S, Lopez W (2012). Expensive Egos: Narcissistic Males Have Higher Cortisol. PLoS ONE.

[CR81] Reuters. 2013. “Swiss Billionaire Gets 18 Years Jail for Italian Asbestos Deaths.” *Reuters*. Available from: https://uk.reuters.com/article/uk-italy-asbestos/swiss-billionaire-gets-18-years-jail-for-italian-asbestos-deaths-idUKBRE9520UZ20130603.

[CR82] Riva P, Lauro R, Leonor L (2015). Reducing Aggressive Responses to Social Exclusion Using Transcranial Direct Current Stimulation. Social Cognitive and Affective Neuroscience.

[CR83] Røe O, Stella G (2015). Malignant Pleural Mesothelioma: History, Controversy and Future of a Manmade Epidemic. European Respiratory Review.

[CR84] Rösler A, Witztum E (1998). Treatment of Men with Paraphilia with a Long-Acting Analogue of Gonadotropin-Releasing Hormone. New England Journal of Medicine.

[CR85] Sampson R, Laub J, Thornberry TP (1997). A Life Course Theory of Cumulative Disadvantage and the Stability of Delinquency. Development Theories of Crimes and Delinquency.

[CR86] Schiermeier, Q. 2015. “The Science Behind the Volkswagen Emissions Scandal.” *Nature News*. Available from: https://www.nature.com/news/the-science-behind-the-volkswagen-emissions-scandal-1.18426. Accessed 9 Aug 2018.

[CR87] Schlegel K (2000). Transnational Crime: Implications for Local Law Enforcement. Journal of Contemporary Criminal Justice.

[CR88] Siever L (2008). Neurobiology of Aggression and Violence. American Journal of Psychiatry.

[CR89] Simpson S, Piquero N (2002). Low Self-Control, Organizational Theory, and Corporate Crime. Law & Society Review.

[CR90] Singh I, Sinnott-Armstrong WP, Savulescu J (2013). Bioprediction, Biomarkers, and Bad Behavior: Scientific, Legal, and Ethical Challenges.

[CR91] Smith D, Pass HI, Vogelzang N, Carbone M (2005). The History of Mesothelioma. Malignant Mesothelioma.

[CR92] Spiegel. 2011. “Inside the Dioxin Scandal: The Criminal Machinations of the Feed Industry.” *Spiegel*. Available from: http://www.spiegel.de/international/germany/inside-the-dioxin-scandal-the-criminal-machinations-of-the-feed-industry-a-738610.html. Accessed 14 June 2018.

[CR93] Sutherland E (1940). White-Collar Criminality. American Sociological Review.

[CR94] Tabibnia G, Monterosso J, Baicy K (2011). Different Forms of Self-Control Share a Neurocognitive Substrate. Journal of Neuroscience.

[CR95] Transparency International. 2015. “Stopping Corruption to Stop Poverty.” Available from: https://www.transparency.org/news/feature/stopping_corruption_to_stop_poverty. Accessed 27 July 2018.

[CR96] Transparency International. 2004. “Global Corruption Report 2004: Political Corruption.” Available from: https://www.transparency.org/whatwedo/publication/global_corruption_report_2004_political_corruption. Accessed 22 June 2018.

[CR97] United States v. Siemens Bangladesh. 2008. Cr. No. 08-36-9-RJL.

[CR98] United States v. Thorn. 2013. 317 F.3d 107, 111–117 (2d Cir. 2003).

[CR99] Vazsonyi A, Crosswhite J (2004). A Test of Gottfredson and Hirschi’s General Theory of Crime in African American Adolescents. Journal of Research in Crime and Delinquency.

[CR100] Viding E, Hanscombe K, Curtis J (2010). In Search of Genes Associated with Risk for Psychopathic Tendencies in Children: A Two-stage Genome-wide Association Study of Pooled DNA. Journal of Child Psychology and Psychiatry.

[CR101] Viding E, McCrory E, Singh S, Sinnott-Armstrong WP, Savulescu J (2013). Genetic Biomarker Research of Callous-Unemotional Traits in Children: Implications for the Law and Policy Making. Bioprediction, Biomarkers, and Bad Behavior: Scientific, Legal, and Ethical Challenges.

[CR102] Virkkunen M, De Jong J, Bartko J (1989). Relationship of Psychobiological Variables to Recidivism in Violent Offenders and Impulsive Fire Setters: A Follow-up Study. Archives of General Psychiatry.

[CR103] Wei S (2000). How Taxing Is Corruption on International Investors?. Review of Economics and Statistics.

[CR108] WHO. 2015. “Asbestos.” World Health Organization. Available from: http://www.who.int/ipcs/assessment/public_health/asbestos/en/. Accessed 25 June 2018.

[CR104] Williams K, Paulhus D, Hare R (2007). Capturing the Four-Factor Structure of Psychopathy in College Students Via Self-Report. Journal of Personality Assessment.

[CR105] Wolpe P, Singh S, Sinnott-Armstrong WP, Savulescu J (2013). Rethinking the Implications of Discovering Biomarkers for Biologically-Based Criminality. Bioprediction, Biomarkers, and Bad Behavior: Scientific, Legal, and Ethical Challenges.

[CR106] World Bank. 2017. “Combating Corruption.” Available from: http://www.worldbank.org/en/topic/governance/brief/anti-corruption. Accessed 28 June 2018.

[CR107] World Bank. 1997. “Helping Countries Combat Corruption: The Role of the World Bank.” Available from: http://www1.worldbank.org/publicsector/anticorrupt/corruptn/coridx.htm. Accessed 19 June 2018.

[CR109] Xiu C, Klein K (2010). Melamine in Milk Products in China: Examining the Factors that Led to Deliberate Use of the Contaminant. Food Policy.

